# Characterization of coagulation-related gene signature to predict prognosis and tumor immune microenvironment in skin cutaneous melanoma

**DOI:** 10.3389/fonc.2022.975255

**Published:** 2022-08-18

**Authors:** Binyu Song, Hao Chi, Gaoge Peng, Yajuan Song, Zhiwei Cui, Yuhan Zhu, Guo Chen, Junzheng Wu, Wei Liu, Chen Dong, Yuanyong Wang, Ke Xu, Zhou Yu, Baoqiang Song

**Affiliations:** ^1^ Department of Plastic Surgery, Xijing Hospital, Fourth Military Medical University, Xi’an, China; ^2^ Clinical Medical College, Southwest Medical University, Luzhou, China; ^3^ Department of Thoracic Surgery, Tangdu Hospital of Air Force Military Medical University, Xi’an, China

**Keywords:** coagulation, prognostic signature, SKCM, TCGA, TME

## Abstract

**Backgroud:**

Skin cutaneous melanoma (SKCM) is an extremely metastatic form of skin cancer. However, there are few valuable molecular biomarkers, and accurate diagnosis is still a challenge. Hypercoagulable state encourages the infiltration and development of tumor cells and is significantly associated with poor prognosis in cancer patients. However, the use of a coagulation-related gene (CRG) signature for prognosis in SKCM, on the other hand, has yet to be determined.

**Method:**

We used data from The Cancer Genome Atlas (TCGA) and Genotype Tissue Expression (GTEx) databases to identify differentially expressed CRGs, then designed a prognostic model by using the LASSO algorithm, univariate and multivariate Cox regression analysis, and constructed a nomogram which was evaluated by calibration curves. Moreover, the Gene Expression Omnibus (GEO), GSE54467 was used as an independent validation. The correlation between risk score and clinicopathological characteristics, tumor microenvironment (TME), and immunotherapy was further analyzed.

**Results:**

To develop a prognostic model, seven CRGs in SKCM patients related to overall survival (OS) were selected: ANG, C1QA, CFB, DUSP6, KLKB1, MMP7, and RABIF. According to the Kaplan-Meier survival analysis, an increased OS was observed in the low-risk group than in the high-risk group (P<0.05). Immunotherapy was much more beneficial in the low-risk group, as per immune infiltration, functional enrichment, and immunotherapy analysis.

**Conclusions:**

The prognosis of SKCM patients may now be predicted with the use of a CRG prognostic model, thus guiding the development of treatment plans for SKCM patients and promoting OS rates.

## Introduction

Skin cutaneous melanoma (SKCM) is an extremely invasive and deadliest form of cancer, resulting in 55,500 deaths each year and accounting for approximately 80% of deaths related to skin cancer (1, 2). Currently, the most common treatment options for melanoma include surgery, chemotherapy, radiotherapy, and immunotherapy (3), but an increased incidences of relapse have emerged as a poor prognostic factor for melanoma (4). In recent years, gene expression is emerging as a promising biomarker and therapeutic target for human cancers. As a result, there is a pressing need to identify appropriate SKCM biomarkers for predicting SKCM prognosis and to carry out personalized therapeutic regimens.

The coagulation system is an innate defense mechanism that is activated either by an extrinsic (tissue factor pathway) or intrinsic pathway. It has been found that tumor cells can express procoagulant factors, such as tissue factor, which trigger the coagulation cascades leading to thrombin production (5–7). There are many coagulation abnormalities in cancer patients, which provide the background for the increased trend of thrombosis and bleeding in these patients. The activation of coagulation and fibrinolysis interacts directly with malignancy by promoting tumor cell invasion, progression, induction of angiogenesis, and ultimately poor prognosis (8). One study reported that cancer patients, especially those with a better prognosis, had significantly a longer survival time with anticoagulant therapy (9). Many biomarkers related to coagulation disorders are confirmed to be significantly related to prognosis in various cancers (10–13). The impact of coagulation on tumors has become an area of intense research interest. However, the role of coagulation in SKCM is still not clearly understood.

The goal of this research was to reveal the potential characteristics of CRGs in SKCM patients, investigate its association with SKCM patients’ prognosis, and then provide an alternative candidate tool to detect SKCM, predict patients’ prognosis, and facilitate clinical management. We identified differential CRGs significantly associated with SKCM patient prognosis by differential gene analysis and univariate Cox regression analysis, and further constructed a 7-gene prognostic model (ANG, C1QA, CFB, DUSP6, KLKB1, MMP7, RABIF) by LASSO algorithm and multivariate Cox regression analysis. The link between the genetic features and the tumor microenvironment, as well as immunotherapy, was also comprehensively investigated. The susceptibility of SKCM patients towards immunotherapy might predictably be assessed using this new signature.

## Materials and methods

### Data collection

The TCGA (http://gdc.cancer.gov) database was used to retrieve and evaluate the mRNA expression profiles, clinical data, and mutation data of SKCM patients. In order to raise the normal sample size, the Genotype-Tissue Expression (GTEx) database was utilized to collect expression profiles of 556 normal skin samples. Patients from the TCGA-SKCM (n=471) served as the internal training set, while the GSE54467 dataset (n=79) from the GEO database was utilized for external validation. R “Limma” package was employed to process and merge data collected from GTEx and TCGA.

### Selection of CRGs in SKCM

The Molecular Signature Database (MsigDB) was utilized for selecting 139 CRGs in total (**Supplementary Table S1**). The “limma” R package was employed for performing the differential genes (DEGs) analysis among the normal and tumor samples, applying |log2FC|≥ 1 and FDR<0.05 as criteria (14). For selecting the genes related to prognosis in SKCM patients, the correlation among all the CRGs and OS, with a threshold defined as p<0.05 was analyzed employing the univariate Cox analysis. Overlapping genes between DEGs and prognosis-associated CRGs were considered as candidate CRGs, which were visualized by Venn diagram package.

### Prognostic model construction based on CRGs

We selected hub genes from candidate CRGs and constructed a novel prognostic model for SKCM *via* LASSO and multivariate Cox regression analysis. Each SKCM patient’s risk score was computed as *Risk score = sum (each gene’s expression × corresponding coefficient)*. The “survival” and “survminer” R packages were used to generate Kaplan-Meier survival curves for the two groups. The prognostic performance of the signatures was evaluated using the independent external validation set, GSE54467 and the concordance index (C-index) and time-dependent ROC curve. Moreover, multivariate and univariate Cox regression analyses were employed to confirm whether risk score and clinicopathological features (including age, stage, and gender) might be independent predictors of SKCM prognosis. Based on the analysis of independent prognostic factors and through the use of R package “rms”, age, stage, and risk scores were utilized for constructing a nomogram to anticipate the prognosis of SKCM patients, and calibration curves were applied for assessing the nomogram’s prognostic accuracy.

### Functional enrichment analysis

Using the R “clusterProfiler” and “circlize” packages, we performed a differential analysis of gene expression profiles between high- and low-risk groups, and a Gene Ontology (GO) functional enrichment analysis for differential genes (|log2FC|≥ 1 and FDR< 0.05) between the two risk subgroups. GO analysis included cellular component (CC), molecular function (MF), biological process (BP), and pathway analysis. Kyoto Encyclopedia of Genes and Genomes (KEGG) analysis on high- or low-risk groups was conducted by Gene Set Variation Analysis (GSVA) by employing the R “GSVA” package, pathways with p-adj < 0.05 were regarded as considerably enriched.

### Correlation analysis among risk score and TME

By utilizing seven algorithms that included TIMER (15), CIBERSORT, CIBERSORT-ABS (16, 17), EPIC (18), QUANTISEQ (19), XCELL (20), and MPC-COUNTER (21), the ssGSEA algorithm was used to determine the differences in immune cell infiltration and immune function between the high-risk and low-risk groups, and the results were shown graphically as a heatmap. To predict the percentage of immune-stromal components in TME. The three scores, which included the stromal, ESTIMATE, and immune scores, were calculated using the “ESTIMATE” R package. The immune cell infiltration and the presence of stroma in the TME were assessed *via* immune and stromal scores. ESTIMATE score was employed for assessing the sum of the stromal and immune scores. We also calculated the link between the risk score and the immunological checkpoints.

### Predicting immunotherapy response

An immunophenoscore (IPS) was generated in each sample from the Cancer Immunome Atlas (TCIA) database, a distinguished predictor of anti-PD-1 and anti-CTLA-4 response, and then compared IPS across risk groups in TCGA-SKCM for exploring the relationship between the risk score and IPS. The tumor immune dysfunction and exclusion (TIDE) algorithm was applied for predicting the patients’ response to immunotherapy by calculating the TIDE score as a surrogate biomarker. The SubMap module of GenePattern was applied to validate the reliability of TIDE prediction and for predicting the response of SKCM samples to immune checkpoint blockade.

### Interpreting mutational landscape in the genome

The TCGA was mined for information on mutations in patients with SKCM. R “maftools” package was used to evaluate the top 20 mutated genes and generate oncoplots, providing a visual representation of the mutational landscape in high- and low-risk cohorts.

### Genome-wide analysis of gene

GSCALite provides an online genomic cancer analysis platform by integrating 33 forms of cancer from TCGA and normal tissue genomics data from GTEx (22). In this study, we analyzed the genomic level, copy number level, methylation level, and other different histological levels of CRGs in SKCM by GSCALite and analyzed the pathway activity.

### CRG risk model comparison to other four models

Four models that were constructed according to the gene expression data were chosen to compare the CRGs prognostic model’s performance to that of other existing SKCM prognostic models. These four models include the nine m1A-, m5C- and m6A-related gene signatures developed by Wu et al. (23), the nine ferroptosis-related gene signatures developed by Chen et al. (24), the two ferroptosis-related gene signature developed by Zeng et al. (25), and eight pyroptosis-related gene signature developed by Ju et al. (26). To ensure comparability of the models, we used the same method for computing the risk scores of every SKCM sample in the TCGA dataset. In addition, the samples were categorized into two groups, i.e., high and low based on the median risk values. The overall survival (OS) difference between the two was then evaluated using the log-rank test, and the ROC of each model was calculated. Furthermore, we compared the five models by concordance index (C-index).

### HPA database validation and genetic prognostic value

The HPA database was employed to validate the CRGs expression in SKCM and normal tissues (27), and the R packages “survminer” and “survival” were utilized for showing the CRGs expression at both high and low levels. The prognostic accuracy of CRGs was demonstrated by drawing KM survival curves between two groups.

### Tumor Immune Single Cell Hub database

The Tumor Immune Single-Cell Hub (TISCH; http://tisch.comp-genomics.org) is a 156 large-scale online database of single-cell RNA-seq focused on the TME. In this study, the TISCH database was used to investigate the expression of seven CRGs in cutaneous melanoma TME.

## Results

### Prognostic coagulation-related DEGs identification

**Figure 1:**
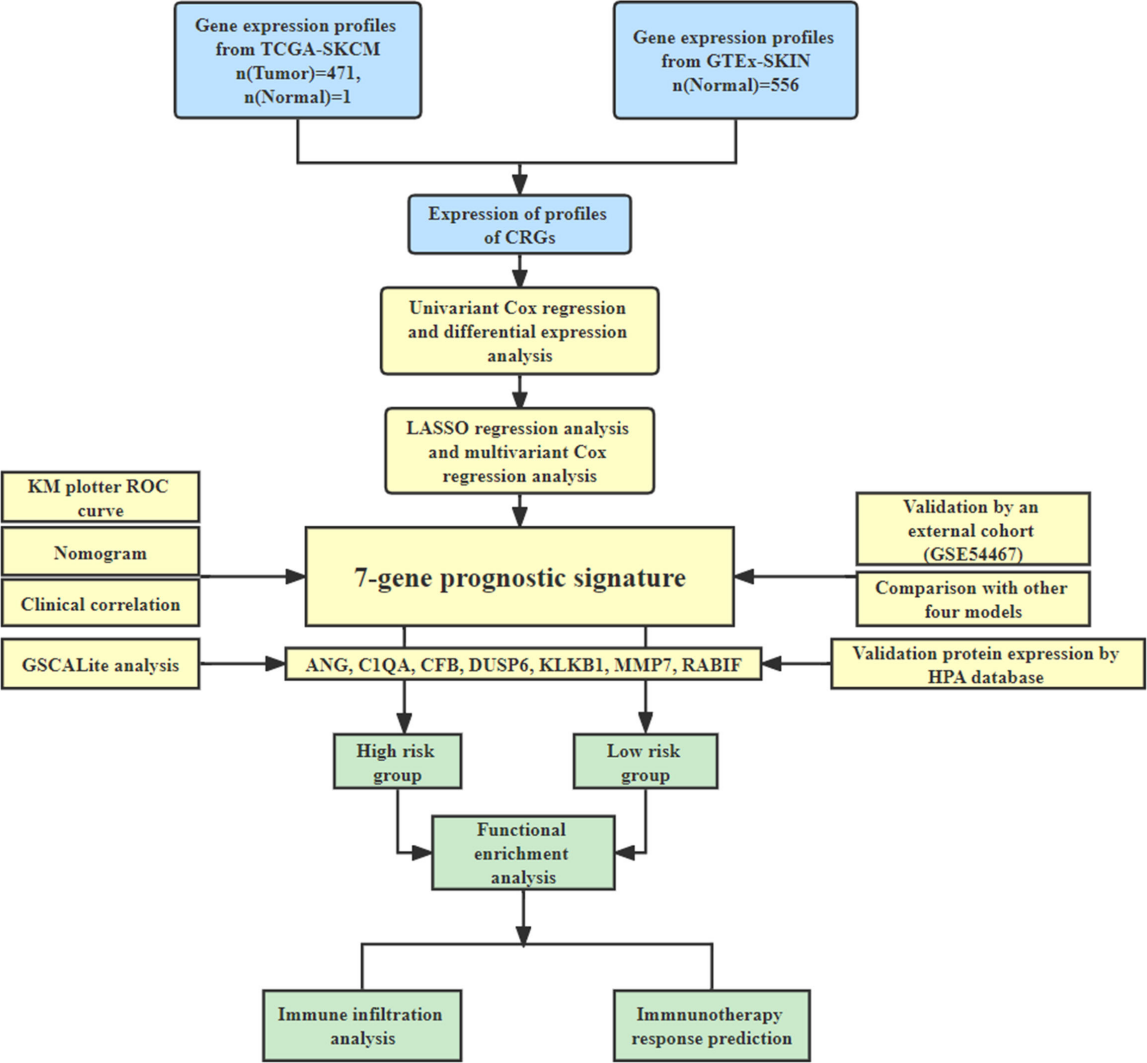
The study workflow.

This study’s workflow is depicted in **Figure 1**.

**Figure 2:**
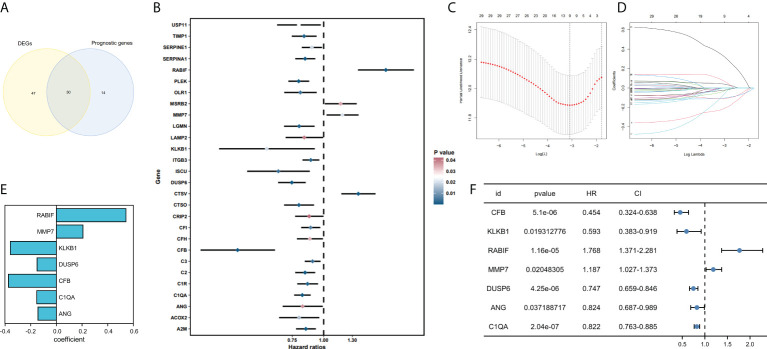
Development and validation of coagulation-related genes predictive signature. **(A)** Intersection of DEGs and Prognostic genes depicted *via* Venn diagram. **(B)** Univariate Cox regression analysis of predictive DEGs related to SKCM survival presented as a forest map. **(C)** LASSO regression of 9 coagulation-related genes. **(D)** Cross-validation for tweaking the parameter in the LASSO regression. **(E)** The corresponding coefficients for 7 candidate genes. **(F)** Univariate Cox regression analysis of 7 candidate genes related to SKCM survival presented as a forest map.

77 genes that were considerably different between the tumor and normal tissues were obtained by comparing the expression profiles of 139 CRGs among TCGA-SKCM and GTEx normal tissues. By analyzing 44 CRGs associated with OS by univariate Cox regression, Venn diagrams revealed 30 differential genes with prognostic values (**Figure 2A**), and outcomes of prognostic analysis of above genes were shown by forest plots (**Figure 2B**).

### Constructing a prognostic model based on the CRGs in SKCM

**Figure 3:**
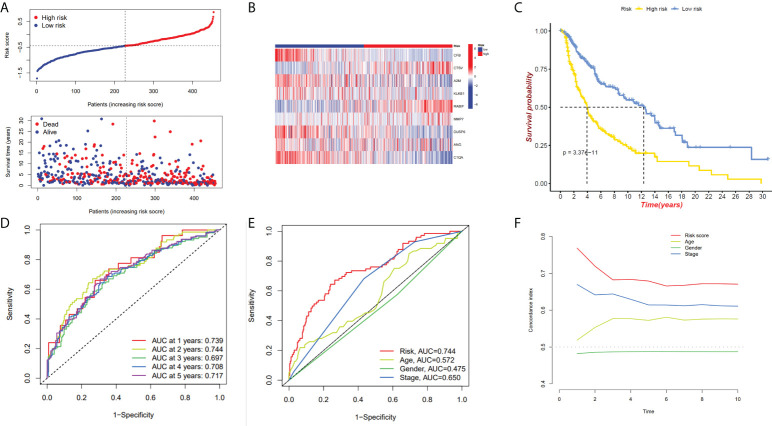
The 7-gene model in the TCGA cohort prognostic analysis. **(A, B)** Risk score distribution and the relevant survival data and the TCGA cohort’s heat map of prognostic genes expression. **(C)** The Kaplan–Meier curve analysis of the TCGA cohort’s high and low-risk groups. **(D)** The AUCs under ROC curves for 1-, 3-, and 5-year OS predictions based on the risk model. **(E)** ROC relating risk scores with clinical indicators of pathology. **(F)** C-index comparison between risk score and clinical features (age, gender, and stage).

**Figure 4:**
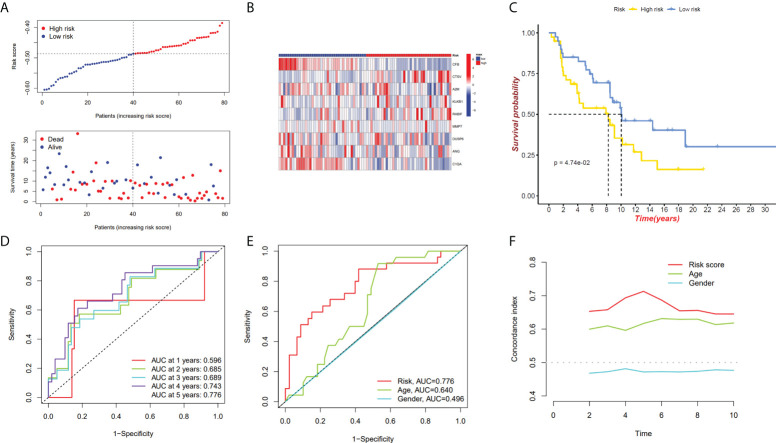
GEO cohort validate prognostic model. **(A, B)** GEO cohort risk score distribution, survival data, and gene expression heat map. **(C)** The GEO cohort’s high-risk and low-risk Kaplan–Meier curves. **(D)**The risk model’s 1-, 3-, and 5-year OS AUCs under ROC curves. **(E)** ROC linking risk scores to pathological markers. **(F)** C-index comparison between risk score and clinical feature (age and gender).

**Figure 5:**
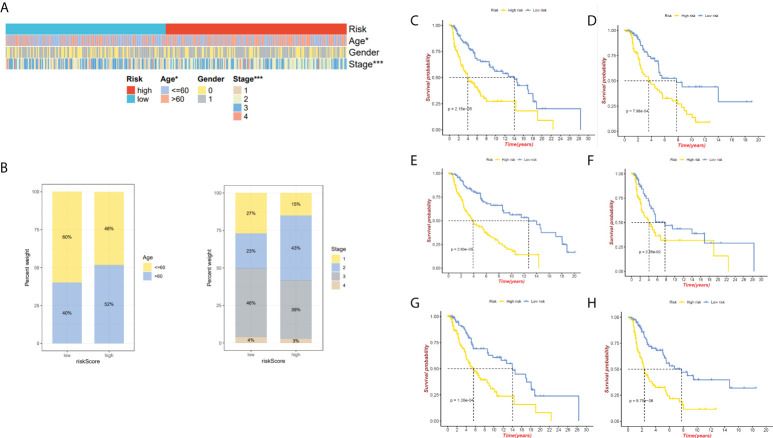
Relationship between both the risk model and clinical features. **(A)**The heatmap presents the distribution of clinicopathological features in two risk subgroups. **(B)** Risk score distribution stratified by age and stage. **(C)** The K-M curve of age ≤ 60 between high- and low-risk groups. **(D)**The K-M curve of age>60 between high- and low-risk groups. **(E)** The K-M curve of male between high- and low-risk groups. **(F)** The K-M curve of female between high- and low-risk groups. **(G)**The K-M curve of stages I&II between high- and low-risk groups. **(H)** The K-M curve of stages III&IV between high- and low-risk groups. *P < 0.05 and ***P < 0.001.

We have confirmed a total of 30 differential genes of prognostic values, utilized the TCGA cohort as the training set, extracted 9 genes by further narrowing the scope of gene screening through the LASSO Cox regression algorithm, and chose the penalty parameter according to the minimum criterion (**Figure 2C, D**). Multivariate Cox regression analysis was performed on the 9 genes screened by LASSO, and finally, 7 genes were selected to construct the prognostic model. **Figure 2E** shows the corresponding coefficients of these 7 genes. A univariate Cox regression analysis was done to determine 7 candidate genes (**Figure 2F**). GEO cohort was utilized as an external validation set for verifying the model’s accuracy. Risk curve, survival state distribution and risk score heatmap of the TCGA cohort and GEO cohort are presented in **Figures 3A, B**, **Figures 4A, B**. The K-M survival curves showed that low-risk patients scored higher than high-risk patients in terms of OS (**Figure 3C**, **Figure 4C**). The specificity and sensitivity of the prognostic model, which had AUCs of 0.739, 0.744, 0.679, 0.708, and 0.717 at years 1, 2, 3, 4, and 5, respectively, in the TCGA cohort, were assessed using time-dependent ROC analysis (**Figure 3D**). The AUCs for the validation dataset were 0.596, 0.685, 0.689, 0.743, and 0.776 at year 1, 2, 3, 4, and 5, respectively (**Figure 4D**). Multiple ROC curves and C-index analysis based on risk score and clinicopathologic characteristics for both cohorts also showed that the AUC and C-index of the risk score were higher than other clinical indicators, suggesting that the risk score may serve as a candidate predictor of prognosis for SKCM patients (**Figures 3E, F**, **Figures 4E, F**). The above results suggest that our model has good prognostic predictive efficacy. It was found that high-age patients and high-stage patients had higher risk scores (**Figures 5A, B**), and to validate the prognostic model’s predictive capabilities for patients with different clinical characteristics in the subgroup analysis, the survival analysis was executed for both high- and low-risk groups with different clinical characteristics, different age groups (≤60 and >60) (**Figures 5C, D**), different gender groups (male and female) (**Figures 5E, F**), and different stage groups (I + II and III + IV) (**Figures 5G, H**) had significantly different prognosis, and the high-risk group patients had a shorter survival time than the low-risk group.

### Construction of the predictive nomogram in SKCM

**Figure 6:**
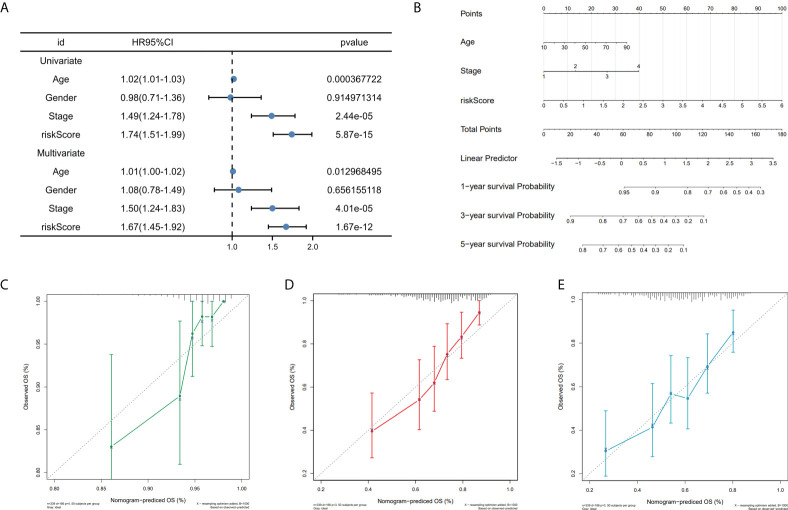
Nomogram construction. **(A)** Univariate and multivariate cox regression prognostic markers. **(B)** Nomogram predicting SKCM patients’ 1-, 3-, and 5-year OS. **(C-E)**. Calibration curves for 1-, 3-, and 5-year patient survival.

To investigate the prognostic model’s independent predictive power, univariate and multivariate Cox regression analysis of clinical characteristics and risk score was performed. The results showed that the risk score, age, and stage had independent prognostic value (**Figure 6A**). Nomogram for predicting 1, 3, and 5-year OS probability in SKCM patients was established, with age, stage, and risk score included as predictors (**Figure 6B**), and the calibration curve showed that the nomogram performs admirably in predicting the probability of survival in SKCM patients (**Figures 6C, D, E**).

### Functional enrichment analysis

**Figure 7:**
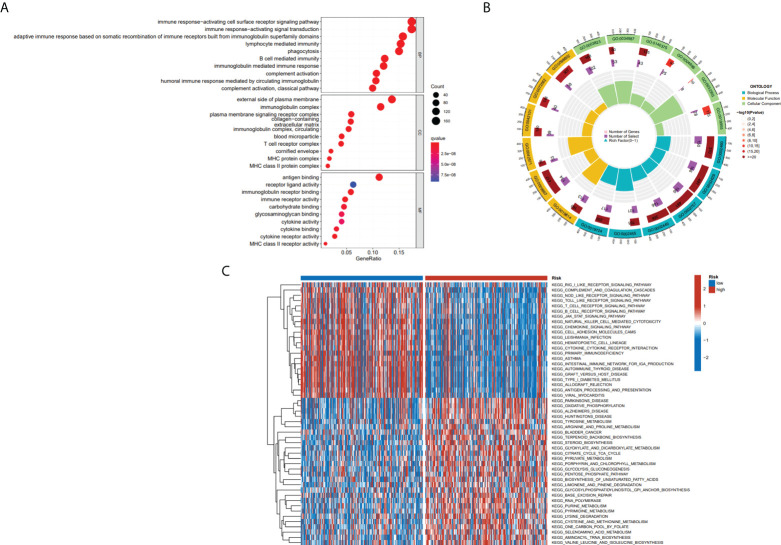
The results of functional analyses. **(A, B)** Gene ontology (GO) enrichment analysis. **(C)** KEGG pathway enrichment by GSVA between two risk subgroups.

To further define the physiological functions and pathways associated with risk scores, a GO enrichment analysis of DEGs between the high- and low-risk groups was conducted, and the findings showed that DEGs between the two groups were largely enriched in immune-related pathways, which include B cell-mediated immunity, immune response-activating signal transduction and immune response-activating cell surface receptor signaling pathway (**Figures 7A, B**). After we further performed GSVA analysis of potential biological roles in both high- and low-risk groups, the outcomes reveal that 75 pathways were considerably different between subgroups (adj p-value < 0.05; **Supplementary Table S1**). The high-risk group was mainly enriched in pathways such as Alzheimers disease, Parkinsons disease, citrate cycle tca cycle, RNA polymerase, and some metabolism pathways. The low-risk group was considerably enhanced in the T cell receptor signaling pathway, natural killer cell-mediated cytotoxicity, B cell receptor signaling pathway, antigen processing, and presentation, and other immune-related pathways (**Figure 7C**). We were surprised to find that both GO terms and KEGG pathways were associated with immunity. Therefore, the link between immune response and risk score was further studied.

### Immune cell infiltration and tumor microenvironment analysis in risk subgroups based on CRGs

**Figure 8:**
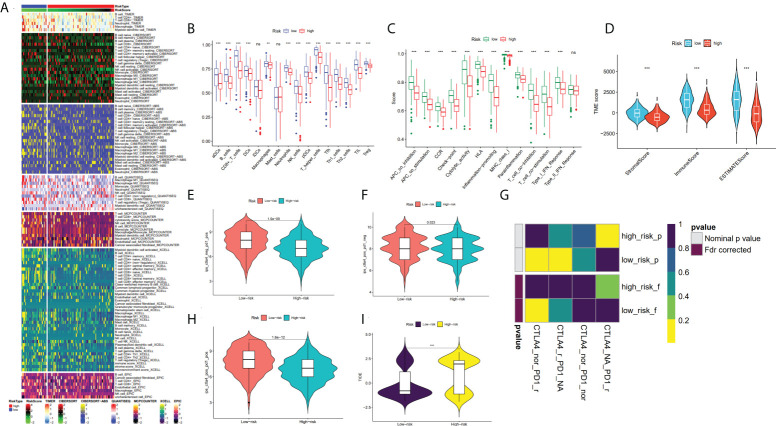
Analysis of TME and immunotherapy among the high- and low-risk groups with SKCM patients. **(A)** Heatmap for immune infiltration based on QUANTISEQ, TIMER, CIBERSORT-ABS, CIBERSORT, XCELL, MCP-counter, and EPIC algorithms among high- and low-risk groups. **(B, C)** Based on the ssGSEA algorithm, the box plots illustrated the variation in immune cell infiltration and immune function among the low- and high-risk groups of SKCM patients. **(D)** Comparison of the immune, stromal and ESTIMATE scores between the low- and high-risk groups, respectively. **(E, F, H)** The distribution of IPS in the high-risk and low-risk groups in TCGA dataset. **(I)** The TIDE score between two risk subgroups. **(G)** SubMap analysis manifested that the low-risk group was more sensitive to the anti-PD-1 therapy. (*P < 0.05, **P < 0.01, ***P < 0.001 and ns = not significant).

The immune infiltration heatmap as per the TIMER, MCPCOUNTER, CIBERSORT-ABS, QUANTISEQ, XCELL, CIBERSORT, and EPIC tools was demonstrated in **Figure 8A**. Utilizing the ssGSEA algorithm, infiltration of immune cells and function were investigated in the TCGA cohort, finding that practically all immune cell types and functions, along with pathways, were considerably increased in low- as to that of high-risk group (p < 0.05; **Figures 8B, C**), and using the ESTIMATE technique, we discovered that low-risk individuals had higher immune, stromal, and estimate scores than high-risk individuals (p < 0.001; **Figure 8D**). Studies have revealed that the advent of immune checkpoint inhibitor (ICIs) therapy offers great promise in clinically treating human cancers (28, 29). Therefore, the correlation between the immune checkpoint molecule (ICM) expression and risk score must be analyzed. It was found that ICM and risk score showed a negative correlation, except for VTCN1, TNFRSF14, and CD276 (**Supplementary Figure S1**). Therefore, it is suggested that patients in low-risk groups could be better candidates for immunotherapy.

### Analysis of immunotherapy between risk subgroups

In recent years, anti-CTLA4 and anti-PD1 have significantly improved the prognosis of late melanoma and increased the OS of patients in tumor immunotherapy (PMID: 34509219). We used the TCIA database to generate IPS for each SKCM sample, and low-risk patients had greater IPS for anti-CTLA-4, anti-PD-1, and anti-(CTLA-4 plus PD-1) than high-risk patients, suggesting better immunotherapy outcomes in patients with lower risk score (**Figures 8E, F, H**). By applying the TIDE algorithm, the TIDE score in the high-risk group was discovered to be substantially greater, implying that low-risk patients responded to immunotherapy in a much more effective manner than the ones in the high-risk group (p < 0.001; **Figure 8I**). Additionally, SubMap analysis demonstrated that the low-risk group would probably respond better to anti-PD-1 therapy (p < 0.05; **Figure 8G**). These findings imply that low-risk individuals may benefit more from immunotherapy, particularly anti-PD-1 treatment.

### Analysis of cancer-related gene mutations in CRG signature

**Figure 9:**
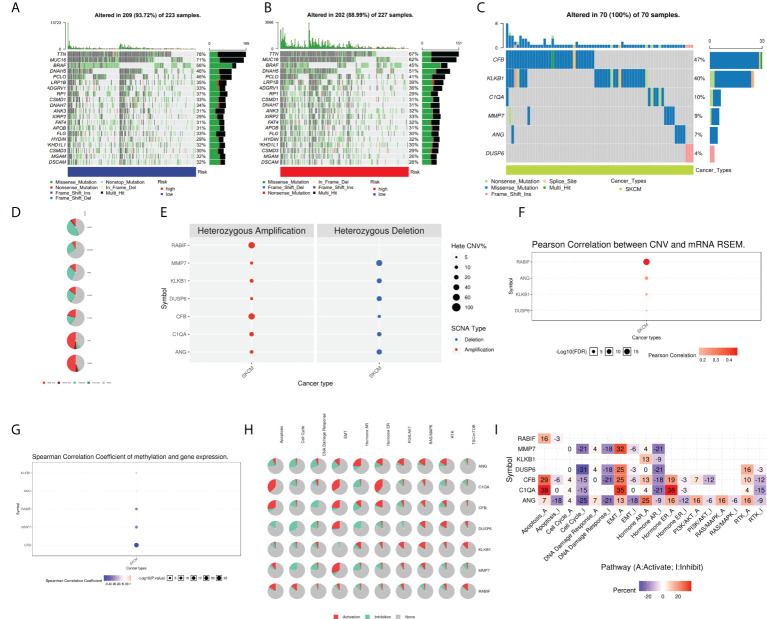
Analysis of ANG, CIQA, CB, DUSP6, KLKB1, MMP7, and RABIF using GSCAlite. **(A)** The oncoplot, commonly called the waterfall plot, depicts the distribution of mutations and SNV types’ classification (which includes missense mutations, frameshift deletions, nonsense mutations, and so on) for the seven genes in the patient samples of SKCM. The side bar graph and top bar graph present the number of mutations in each sample or gene. **(B, C)** Oncoplots display the somatic landscape of SKCM with high- and low-risk group. **(D)** Heterozygous CNV profile presents the percentage of heterozygous CNVs, including the percentage of heterozygous amplification and deletion of these genes in SKCM. **(E)** CNV pie distribution. **(F)** Relationship between CNV and gene expression. Red bubbles demonstrate positive correlation, meaning that the gene expression will become upregulated when genes have high frequency of CNV. The darker the color, the higher the correlation. The size of the dots demonstrates statistical significance: the statistical significance increases as the sample size grows. **(G)** Correlation analysis among the gene expression levels and methylation. Down-regulated methylation in tumors is represented by blue dots, while up-regulated methylation in tumors is represented by red dots. **(H)** Pie-shaped distribution of the above 7 genes in cancer pathway, red depicts activation, green represents suppression; **(I)** Percentages of these genes in cancer pathway (I depicts suppression; A depicts activation), darker color depicts higher percentage. SNV, single nucleotide variation. CNV, copy number variation.

Moreover, the oncoplots summarized high- and low-risk mutations (**Figures 9A, B**). Among them, TTN (76% vs 67%), MUC16 (71% vs 62%), and BRAF (56% vs 45%) were the most common, exerting a greater somatic mutation frequency in low-risk groups than in the high-risk group. To further investigate the biological mechanisms underlying the aberrant expression of these seven target genes in our model, these CRGs were analyzed at different histological levels, including genomic level, copy number level, and methylation level. The results showed that CFB was the gene with an extremely high rate of mutation, while DUSP6 had the lowest mutation rate (**Figure 9C**). Subsequently, we analyzed CNV data in TCGA and found that CNVs in SKCM patients included heterozygous amplification and deletion (**Figure 9D**). Analysis of CNV percentages for Hete Amp and Hete Del revealed that the Hete Amp levels of RABIF and CFB in SKCM were greater than 60%. Meanwhile, MMP7 exhibits more than 60% Hete Del (P < 0.05) (**Figure 9E**). Additional analysis into the relationship between these genes’ expression levels and CNV showed that DUSP6, KLKB1, ANG, and RABIF were positively correlated with CNV, with RABIF being the most significantly correlated with CNV (**Figure 9F**). In addition, Spearman correlation coefficient analysis among the methylation and gene expression was carried out in the methylation difference bubble plots, and it was found that the methylation of ANG, CFB, MMP7, and RABIF was down-regulated in SKCM, while KLKB1 was up-regulated (**Figure 9G**), and the abnormal gene expression might be the result of the combined effect of copy number variation and methylation variation. These seven genes mostly control apoptosis, cell cycle, EMT, DNA damage response, and other tumor-related processes, as demonstrated by pathway analysis **(** **Figure 9H** **)**. The activated and inhibited pathways were labeled as A and I, as illustrated in **Figure 9I**, to further evaluate if each pathway is activated or repressed. It can be seen that under the regulation of seven genes, RAS/MAPK and Hormone ER pathways were activated, cell cycle and DNA damage response were inhibited, while apoptosis, EMT, Hormine AR and PI3K/AKT pathways were activated or inhibited in SKCM patients.

### Comparison of the CRG prognostic risk model with other existing models

**Figure 10:**
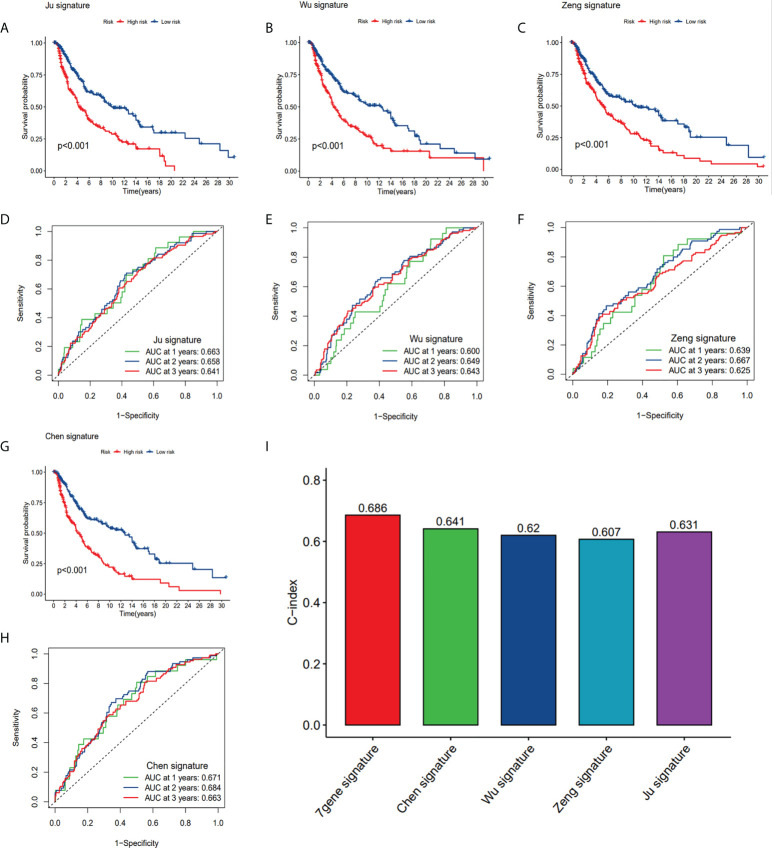
Comparison of CRG risk model with others. **(A, D)** The KM curves and ROC of an eight-gene (Ju) risk model. **(B, E)** The KM curves and ROC of a nine-gene (Wu) risk model. **(C, F)** The KM curves and ROC of a ten-gene (Zeng) risk model. **(G, H)** The KM curves and ROC of a nine-gene (Chen) risk model. **(I)** C-indexes of the five risk models.

In order to assess whether our seven-gene signature has significant predictive power, it was compared with the four published prognostic signatures of SKCM, which are: the eight pyroptosis-associated gene signature (Ju et al.), the nine ferroptosis-related gene signature (Chen et al.), the nine m1A-, m5C- and m6A-related gene signature (Wu et al.), and the two ferroptosis-related gene signature (Zeng et al.). The same approach was used to calculate SKCM patients’ risk score according to their modelled genes to compare signatures, the KM curves and ROC curves of the respective models. Survival analyses were considerably varied for the high- and low-risk subgroups of all four signatures (**Figures 10A-C, G**). However, ROC curve analysis showed higher AUC values in our CRGs model than other prognostic models in 1, 2, and 3-year survival (**Figures 10D** **-** **6F**, **10H**). By computing this model’s and four others’ C-indices, the results showed that the C-index of 0.686 of our model was the greatest (**Figure 10I**). Our gene signature’s remarkable predictive capabilities were emphasized by such outcomes.

### Protein expression level validation of the screened genes by HPA database

**Figure 11:**
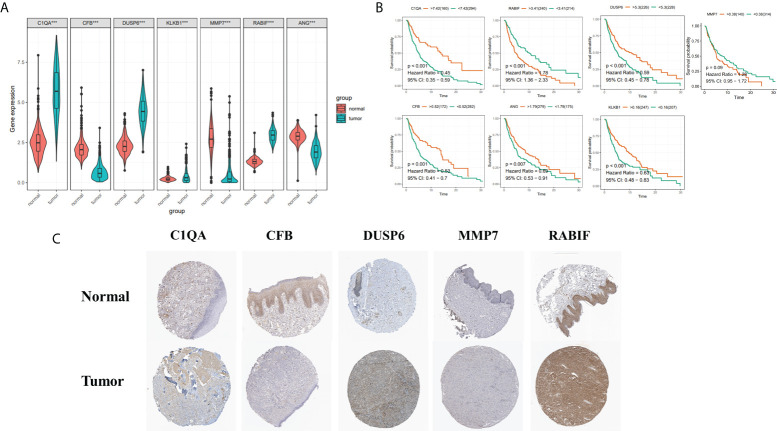
**(A)** 7 Hub genes in normal GTEx, TCGA, and TCGA cancer tissues. **(B)** KM curves for 7 high- and low-expression hub genes, ordinal (y-axis) indicates percentage of survival, abscissa (x-axis) represents survival years **(C)** HPA database hub gene validation.

To boost the database’s credibility, 7 CRGs’ protein expression was analyzed using the HPA database. Among them, ANG and KLKB1 were not collected in the HPA database (**Figure 11C**). These results are consistent with our analysis of differential gene expression (**Figure 11A**). Kaplan-Meier curves of five genes (C1QA, DUSP6, RABIF, MMP7, CFB) in HPA database were shown in **Supplementary Figure S3**. Kaplan-Meier curves in TCGA database showed that increased RABIF expression was considerably related to low OS in SKCM patients, and increased MMP7 expression may indicate a poorer prognosis in SKCM patients, but lacked statistical significance (**Figure 11B**).

### Correlation analysis of CRGs and TME

**Figure 12:**
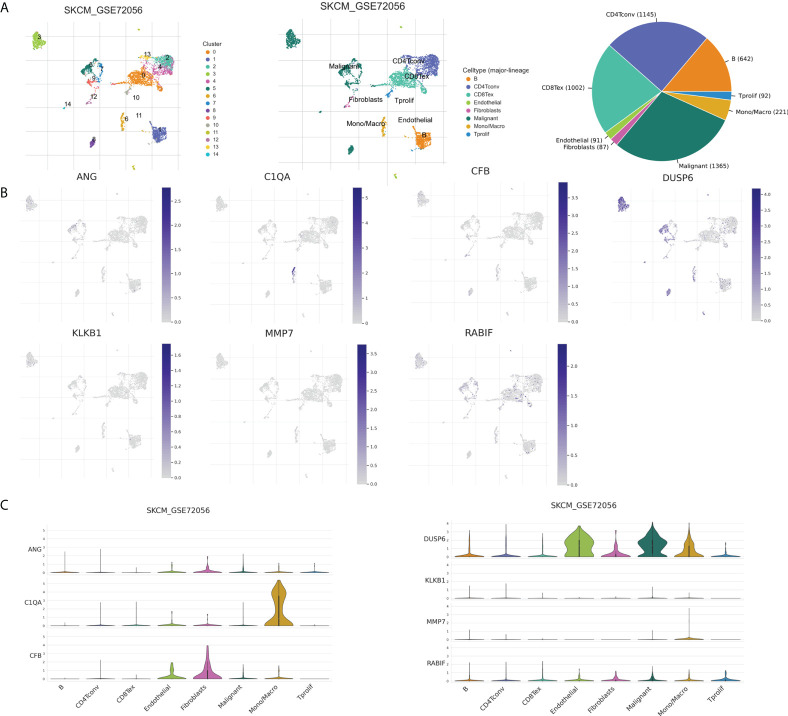
CRGs expression in SKCM TME-associated cells. **(A)** Annotation of diverse cell types and percentages in GSE72056. **(B, C)** Expressions of ANG, C1QA, CFB, DUSP6, KLKB1, MMP7, and RABIF in different cell types in GSE72056.

The tumor microenvironment, including malignant cells, stromal cells, and immune cells, plays an important role in tumorigenesis, progression, and recurrence. Therefore, we used six single-cell datasets (SKCM_GSE115978_aPD1, 281 SKCM_GSE120575_aPD1aCTLA4, SKCM_GSE123139,SKCM_GSE139249, 282 SKCM_GSE148190, and SKCM_GSE72056) to analyze the distribution of the seven model genes in TME-related cells. Among them, ANG and CFB were highly expressed in fibroblasts, C1QA and MMP7 were mainly expressed in macrophages, and KLKB1, DUSP6, and RABIF were mainly expressed in malignant cells, while RABIF was also highly expressed in immune cells, such as proliferating T cells and macrophages, etc(**Figures 12B, C**, **Supplementary Figure S2**). **Figure 12A** visualizes the distributions and numbers of the different cell types in GSE72056, which contains 14 clusters and 8 types.

## Discussion

SKCM is potentially amongst the most lethal skin cancers with a rapidly increasing incidence all over the world and still controversial in pathogenesis (30). Previous study found interactions and effects between malignant tumor progression and coagulation (31). Tumor can be affected by the production of procoagulant factors, including tissue factor (TF), cancer procoagulant proteins, microparticles (MPs), proangiogenic factors, and cytokines, etc. The production and activation of these procoagulant factors further promote tumorigenesis and development, resulting in a chronic hypercoagulable state and an increased incidence of thrombotic events (32). It has been found that elevated plasma fibrinogen in SKCM patients is often associated with decreased survival (33). In addition, high D-dimer levels (the end product of coagulation cascade activation) suggest fibrin lysis and activated coagulation and are related with advanced cancer staging and poor prognosis in SKCM patients (34, 35). Since single prognostic molecular indicators and traditional clinical factors are difficult to assess the clinical outcome of SKCM, an urgent requirement exists to develop an accurate model to predict survival of the patients for facilitating the management of clinical treatment.

In the current research, differential analysis of the expression matrix of coagulation-related mRNAs of SKCM was performed in the TCGA database, and using univariate, LASSO, and multivariate Cox regression analysis, we developed a new prognostic model based on 7 CRGs (ANG, C1QA, CFB, DUSP6, KLKB1, MMP7, and RABIF) and confirmed its exceptional performance in an external population. As per an increasing body of evidence, the CRGs are linked to the prognosis of numerous malignancies. For example, the biological function of KLKB1 is usually associated with coagulation surface-dependent activation, fibrinolysis, and inflammation, and high KLKB1 expression in patients with hepatocellular carcinoma predicts a good prognosis (36). DUSP6 is an oncogenic factor in melanoma, and DUSP4 can play its part in the survival and growth of melanoma cells by inhibiting the DUSP6 expression. Increased RABIF expression in breast and triple negative breast cancer patients is related to poorer outcome and considered a potential bioprognostic marker (37). MMP7 is a stromal lysin protein playing important roles in immunity, apoptosis, angiogenesis, and coagulation and is highly expressed in cancers of various types, including gastric cancer, uveal melanoma, and colon cancer, resulting in infiltration and metastasis of tumor cells (38–40). One study revealed that increased C1QA expression was significantly related to a better prognosis in breast cancer (41). MiR-491-5p was reported to directly targeting RABIF to downregulate cell invasion, metastasis, and drug resistance in triple negative breast cancer (37).

The outcomes of GO analysis demonstrated that cell coagulation genes were shown to be highly linked to functional pathways including B cell-mediated immunity, immune response-activating signal transduction, and immune response-activating cell receptor surface signaling pathway, etc. The B cell, T cell receptor signaling pathwayand natural killer cell-mediated cytotoxicity were all significantly enhanced in the low-risk group, according to GSVA analysis. Therefore, we hypothesized that cellular coagulation genes and risk signature are related to immunity.

Earlier studies revealed that the TME has played a crucial part in SKCM occurrence, progression, and treatment resistance (42). As expected, CRG-based risk signature was found to be considerably associated with the TME of SKCM. To investigate the relation between the risk subgroups and immune cell infiltration levels, eight algorithms were utilized, and it was found that immune cell infiltration levels were elevated in the low-risk group and immune function was adequately performed. The Estimate algorithm’s outcomes also suggested that tumor cells in the low-risk group had a higher immune cell and stromal cell infiltration as compared to the high-risk group, implying that the low-risk group of SKCM had higher immunogenicity and immune response, and the high-risk patients may have significantly reduced anti-tumor immunity. Immune checkpoint inhibitors can kill cancer cells by inducing CD8-positive T cells, especially anti-CTLA4 and anti-PD-1 antibodies that have been widely used in SKCM and revolutionized its treatment (43), and it can be concluded from the previous analysis that the inflammatory features of TME are stronger in the low-risk group. A characteristic of the inflammatory TME is the increased expression of immune checkpoints, and the expression of almost all immunological checkpoints was found to be negatively correlated with risk scores, in this investigation.

According to IPS analysis, low-risk patients had significantly better IPS outcomes than high-risk patients, suggesting that low-risk patients are a better candidate for immune checkpoint inhibitor treatment, and based on machine learning algorithms such as TIDE and Submap, it was surprising to find that the low-risk group was susceptible towards anti-PD-1 but not towards anti-CTLA-4 immunotherapy.

Genome-wide analysis of candidate CRGs showed that these high frequencies of copy number variants and methylation were closely associated with gene expression. Furthermore, these seven genes regulate various tumor-associated pathways, which activate the RAS/MAPK pathway, and the aberrant activation of the RAS/MAPK signaling pathway leads to SKCM cell cycle dysregulation and apoptosis inhibition, which is a central step in SKCM progression. Both the specific regulatory mechanisms of these genes on tumor-associated pathways, and the activation or inhibition of these tumor-associated pathways on SKCM development and tumor cell behavior require more research to explore.

Before us, Wu (23), Ju (26) and Chen (24) constructed risk prognostic signatures for survival prediction in SKCM patients based on m1A-, m5C- and m6A-related genes (MBD4, RBM15B, MBD2, UHRF2, WTAP, YTHDF1, UNG, FMR1, NEIL1), pyroptosis-related genes (GSDMD, GSDME, CASP4, NLRC4, APIP, AIM2, CASP3, IL18), and ferroptosis-related genes (ACSL4, CS, ATP5MC3, ACACA, CHAC1, ALOX5, MT1G, ABCC1, ZEB1), respectively. And Zeng (25) have also built a prognostic model for SKCM based on ferroptosis-related genes (ALOX5, CHAC1). Ju et al. observed that risk score was mainly associated with immunity by functional enrichment analysis of the two risk groups, and other researchers further performed a more comprehensive analysis of tumor immune infiltration and tumor microenvironment of SKCM. The models they constructed demonstrated the importance of ferroptosis, RNA methylation, and pyroptosis-associated genes in predicting the prognosis and TME of SKCM. A prognostic risk model was designed by utilizing coagulation-related genes (ANG, C1QA, CFB, DUSP6, KLKB1, MMP7), the ROC values of model we established indicated that CRG risk model outperformed the four published models above in terms of predictive efficacy. We not only performed immune infiltration and TME analysis, but we also predicted immunotherapy by IPS and TIDE score in high- and low-risk patients. Seven CRGs at different histological levels were explored by using GSCALite, including genomic level, copy number level, and methylation level, and explored their biological mechanisms. Furthermore, the corresponding proteins’ expression levels were investigated through the HPA database to corroborate our analysis. To our knowledge, our work presents a novel coagulation-related gene predictive model for SKCM.

TME consists of malignant cells, stromal cells, and immune cells and plays an important role in tumorigenesis and metastasis (44). We explored the distributions of seven genes through single-cell datasets, and we speculate that ANG, DUSP, and CFB may affect prognosis mainly by regulating stromal cells, such as fibroblasts and malignant cells. C1QA may affect melanoma progression and prognosis by regulating macrophages. KLKB1 may influence prognosis of melanoma patients by regulating malignant cells. RABIF is highly expressed in a range of immune cells and malignant cells, and we speculate that it may affect tumor progression and prognosis by regulating malignant cells and proliferating T cells. However, detailed and precise mechanisms need to be further explored in the future.

Even so, there are certain limitations in our research. Firstly, our observations are needed to be corroborated by multicenter and prospective clinical studies. Second, we demonstrated that seven coagulation-related genes are associated with prognosis in SKCM, but this was evaluated solely by data mining. More investigations are needed to reveal these seven genes’ functions, and the association between coagulation-related genes and the development of SKCM needs to be further explored.

## Conclusion

Lastly, the expression of CRGs is linked to the progression of SKCM patients, according to our findings. A novel coagulation-related prognostic gene signature, which was found to be an independent predictor in SKCM prognosis, was identified including 7 central CRGs. It also reflects the different statuses of the tumor microenvironment between the high and low risk groups and anticipates the potential response to immunotherapy. To our knowledge, this is the first coagulation-related signature to predict prognosis in SKCM patients, providing a new perspective for individualized treatment.

## Data availability statement

The datasets presented in this study can be found in online repositories. The names of the repository/repositories and accession number(s) can be found in the article/**Supplementary Material**.

## Author contributions

Conception and design: BiS and HC. Data curation and methodology: GP, CD, ZC, ZY, and GC. Analysis and interpretation of data: WL, JW, YW, KX and YS. Writing of the manuscript: BiS. Review of the manuscript: ZY and BaS. Study supervision: ZY and BaS. All authors contributed to the article and approved the submitted version.

## Funding

This study was funded by the National Natural Science Foundation of China (82072182) and Shaanxi Science and Technology Coordination and Innovation Project Grants (2020SF-179).

## Acknowledgments

We thank all the members of Mango teamfor generously sharing their experience and codes.

## Conflict of interest

The authors declare that the research was conducted in the absence of any commercial or financial relationships that could be construed as a potential conflict of interest.

## Publisher’s note

All claims expressed in this article are solely those of the authors and do not necessarily represent those of their affiliated organizations, or those of the publisher, the editors and the reviewers. Any product that may be evaluated in this article, or claim that may be made by its manufacturer, is not guaranteed or endorsed by the publisher.

## References

[1] Schadendorf D van Akkooi ACJ Berking C Griewank KG Gutzmer R Hauschild A. Melanoma. Lancet (2018) 392:971–84. doi: 10.1016/S0140-6736(18)31559-9 30238891

[2] Rebecca VW Somasundaram R Herlyn M. Pre-clinical modeling of cutaneous melanoma. Nat Commun (2020) 11:2858. doi: 10.1038/s41467-020-15546-9 32504051 PMC7275051

[3] Reuland SN Goldstein NB Partyka KA Cooper DA Fujita M Norris DA. The combination of BH3-mimetic ABT-737 with the alkylating agent temozolomide induces strong synergistic killing of melanoma cells independent of p53. PLoS One (2011) 6:e24294. doi: 10.1371/journal.pone.0024294 21897876 PMC3163662

[4] Hou M Guo X Chen Y Cong L Pan C. A prognostic molecular signature of N^6^-methyladenosine methylation regulators for soft-tissue sarcoma from the cancer genome atlas database. Med Sci Monit (2020) 26:e928400. doi: 10.12659/MSM.928400 33370249 PMC7780893

[5] Smith SA Travers RJ Morrissey JH. How it all starts: Initiation of the clotting cascade. Crit Rev Biochem Mol Biol (2015) 50:326–36. doi: 10.3109/10409238.2015.1050550 PMC4826570 26018600

[6] Zarà M Canobbio I Visconte C Canino J Torti M Guidetti GF. Molecular mechanisms of platelet activation and aggregation induced by breast cancer cells. Cell Signal (2018) 48:45–53. doi: 10.1016/j.cellsig.2018.04.008 29705335

[7] Hamza MS Mousa SA. Cancer-associated thrombosis: Risk factors, molecular mechanisms, future management. Clin Appl Thromb Hemost (2020) 26:1076029620954282. doi: 10.1177/1076029620954282 32877229 PMC7476343

[8] Korte W. Changes of the coagulation and fibrinolysis system in malignancy: their possible impact on future diagnostic and therapeutic procedures. Clin Chem Lab Med (2000) 38:679–92. doi: 10.1515/CCLM.2000.099 11071061

[9] Tieken C Versteeg HH. Anticoagulants versus cancer. Thromb Res (2016) 140 Suppl 1:S148–153. doi: 10.1016/S0049-3848(16)30114-1 27067969

[10] Kawai K Watanabe T. Colorectal cancer and hypercoagulability. Surg Today (2014) 44:797–803. doi: 10.1007/s00595-013-0606-5 23670036

[11] Tikhomirova I Petrochenko E Malysheva Y Ryabov M Kislov N. Interrelation of blood coagulation and hemorheology in cancer. Clin Hemorheol Microcirc (2016) 64:635–44. doi: 10.3233/CH-168037 27791998

[12] Swier N Versteeg HH. Reciprocal links between venous thromboembolism, coagulation factors and ovarian cancer progression. Thromb Res (2017) 150:8–18. doi: 10.1016/j.thromres.2016.12.002 27988375

[13] Ma M Cao R Wang W Wang B Yang Y Huang Y. The d-dimer level predicts the prognosis in patients with lung cancer: a systematic review and meta-analysis. J Cardiothorac Surg (2021) 16:243. doi: 10.1186/s13019-021-01618-4 34454552 PMC8399789

[14] Diboun I Wernisch L Orengo CA Koltzenburg M. Microarray analysis after RNA amplification can detect pronounced differences in gene expression using limma. BMC Genomics (2006) 7:252. doi: 10.1186/1471-2164-7-252 17029630 PMC1618401

[15] Li T Fu J Zeng Z Cohen D Li J Chen Q. TIMER2.0 for analysis of tumor-infiltrating immune cells. Nucleic Acids Res (2020) 48:W509–14. doi: 10.1093/nar/gkaa407 PMC7319575 32442275

[16] Newman AM Liu CL Green MR Gentles AJ Feng W Xu Y. Robust enumeration of cell subsets from tissue expression profiles. Nat Methods (2015) 12:453–7. doi: 10.1038/nmeth.3337 PMC4739640 25822800

[17] Chen B Khodadoust MS Liu CL Newman AM Alizadeh AA. Profiling tumor infiltrating immune cells with CIBERSORT. Methods Mol Biol (2018) 1711:243–59. doi: 10.1007/978-1-4939-7493-1_12 PMC5895181 29344893

[18] Racle J Gfeller D. EPIC: A tool to estimate the proportions of different cell types from bulk gene expression data. Methods Mol Biol (2020) 2120:233–48. doi: 10.1007/978-1-0716-0327-7_17 32124324

[19] Finotello F Mayer C Plattner C Laschober G Rieder D Hackl H. Molecular and pharmacological modulators of the tumor immune contexture revealed by deconvolution of RNA-seq data. Genome Med (2019) 11:34. doi: 10.1186/s13073-019-0638-6 31126321 PMC6534875

[20] Aran D Hu Z Butte AJ. xCell: digitally portraying the tissue cellular heterogeneity landscape. Genome Biol (2017) 18:220. doi: 10.1186/s13059-017-1349-1 29141660 PMC5688663

[21] Becht E Giraldo NA Lacroix L Buttard B Elarouci N Petitprez F. Estimating the population abundance of tissue-infiltrating immune and stromal cell populations using gene expression. Genome Biol (2016) 17:218. doi: 10.1186/s13059-016-1070-5 27765066 PMC5073889

[22] Liu C-J Hu F-F Xia M-X Han L Zhang Q Guo A-Y. GSCALite: a web server for gene set cancer analysis. Bioinformatics (2018) 34:3771–2. doi: 10.1093/bioinformatics/bty411 29790900

[23] Wu XR Chen Z Liu Y Chen ZZ Tang F Chen ZZ. Prognostic signature and immune efficacy of m ^1^ a-, m ^5^ c- and m ^6^ a-related regulators in cutaneous melanoma. J Cell Mol Med (2021) 25:8405–18. doi: 10.1111/jcmm.16800 PMC8419166 34288419

[24] Chen Y Guo L Zhou Z An R Wang J. Identification and validation of a prognostic model for melanoma patients with 9 ferroptosis-related gene signature. BMC Genomics (2022) 23:245. doi: 10.1186/s12864-022-08475-y 35354376 PMC8969311

[25] Zeng N Ma L Cheng Y Xia Q Li Y Chen Y. Construction of a ferroptosis-related gene signature for predicting survival and immune microenvironment in melanoma patients. Int J Gen Med (2021) 14:6423. doi: 10.2147/IJGM.S327348 34675611 PMC8502037

[26] Ju A Tang J Chen S Fu Y Luo Y. Pyroptosis-related gene signatures can robustly diagnose skin cutaneous melanoma and predict the prognosis. Front Oncol (2021) 11:709077. doi: 10.3389/fonc.2021.709077 34327145 PMC8313829

[27] Uhlen M Oksvold P Fagerberg L Lundberg E Jonasson K Forsberg M. Towards a knowledge-based human protein atlas. Nat Biotechnol (2010) 28:1248–50. doi: 10.1038/nbt1210-1248 21139605

[28] Llovet JM Montal R Sia D Finn RS. Molecular therapies and precision medicine for hepatocellular carcinoma. Nat Rev Clin Oncol (2018) 15:599–616. doi: 10.1038/s41571-018-0073-4 30061739 PMC12452113

[29] Salik B Smyth MJ Nakamura K. Targeting immune checkpoints in hematological malignancies. J Hematol Oncol (2020) 13:111. doi: 10.1186/s13045-020-00947-6 32787882 PMC7425174

[30] Davis EJ Perez MC Ayoubi N Zhao S Ye F Wang DY. Clinical correlates of response to anti-PD-1-based therapy in patients with metastatic melanoma. J Immunother (2019) 42:221–7. doi: 10.1097/CJI.0000000000000258 PMC6561788 30882548

[31] Falanga A Marchetti M Vignoli A. Coagulation and cancer: biological and clinical aspects. J Thromb Haemost (2013) 11:223–33. doi: 10.1111/jth.12075 23279708

[32] García-Escobar I Brozos-Vázquez E Gutierrez Abad D Martínez-Marín V Pachón V Muñoz Martín AJ. Cancer and thrombosis section of the Spanish society of medical oncology (SEOM). direct oral anticoagulants for the treatment and prevention of venous thromboembolism in patients with cancer: current evidence. Clin Transl Oncol (2021) 23:1034–46. doi: 10.1007/s12094-020-02506-4 PMC8084841 33206333

[33] Tas F Ciftci R Kilic L Bilgin E Keskin S Sen F. Clinical and prognostic significance of coagulation assays in melanoma. Melanoma Res (2012) 22:368–75. doi: 10.1097/CMR.0b013e328357be7c 22889867

[34] Desch A Gebhardt C Utikal J Schneider SW. D-dimers in malignant melanoma: Association with prognosis and dynamic variation in disease progress. Int J Cancer (2017) 140:914–21. doi: 10.1002/ijc.30498 27813063

[35] Arce M Pinto MP Galleguillos M Muñoz C Lange S Ramirez C. Coagulation factor xa promotes solid tumor growth, experimental metastasis and endothelial cell activation. Cancers (Basel) (2019) 11:E1103. doi: 10.3390/cancers11081103 31382462 PMC6721564

[36] Che Y-Q Zhang Y Li H-B Shen D Cui W. Serum KLKB1 as a potential prognostic biomarker for hepatocellular carcinoma based on data-independent acquisition and parallel reaction monitoring. J Hepatocell Carcinoma (2021) 8:1241–52. doi: 10.2147/JHC.S325629 PMC8520450 34676182

[37] Huang W-C Chi H-C Tung S-L Chen P-M Shih Y-C Huang Y-C. Identification of the novel tumor suppressor role of FOCAD/miR-491-5p to inhibit cancer stemness, drug resistance and metastasis *via* regulating RABIF/MMP signaling in triple negative breast cancer. Cells (2021) 10:2524. doi: 10.3390/cells10102524 34685504 PMC8534268

[38] Zuidervaart W Pavey S van Nieuwpoort FA Packer L Out C Maat W. Expression of Wnt5a and its downstream effector beta-catenin in uveal melanoma. Melanoma Res (2007) 17:380–6. doi: 10.1097/CMR.0b013e3282f1d302 17992121

[39] Lu L Ma G-Q Liu X-D Sun R-R Wang Q Liu M. Correlation between GDF15, MMP7 and gastric cancer and its prognosis. Eur Rev Med Pharmacol Sci (2017) 21:535–41. 28239815

[40] Chen L Ke X. MMP7 as a potential biomarker of colon cancer and its prognostic value by bioinformatics analysis. Med (Baltimore) (2021) 100:e24953. doi: 10.1097/MD.0000000000024953 PMC7939218 33655961

[41] Azzato EM Lee AJX Teschendorff A Ponder BA Pharoah P Caldas C. Common germ-line polymorphism of C1QA and breast cancer survival. Br J Cancer (2010) 102:1294–9. doi: 10.1038/sj.bjc.6605625 PMC2856004 20332777

[42] Avagliano A Fiume G Pelagalli A Sanità G Ruocco MR Montagnani S. Metabolic plasticity of melanoma cells and their crosstalk with tumor microenvironment. Front Oncol (2020) 10:722. doi: 10.3389/fonc.2020.00722 32528879 PMC7256186

[43] Carlino MS Larkin J Long GV. Immune checkpoint inhibitors in melanoma. Lancet (2021) 398:1002–14. doi: 10.1016/S0140-6736(21)01206-X 34509219

[44] Arneth B. Tumor microenvironment. Medicina (Kaunas) (2019) 56:E15. doi: 10.3390/medicina56010015 31906017 PMC7023392

