# Supplementary material for: Characterization of coagulation-related gene signature to predict prognosis and tumor immune microenvironment in skin cutaneous melanoma
Source: Front Oncol. 2022 Aug 18;12:975255. doi: 10.3389/fonc.2022.975255 (PMC9434152; doi:10.3389/fonc.2022.975255)
Supplement: Supplementary file 1 [file DataSheet_1.docx]

Supplementary Material

# Supplementary Figures

## Supplementary Figures

**
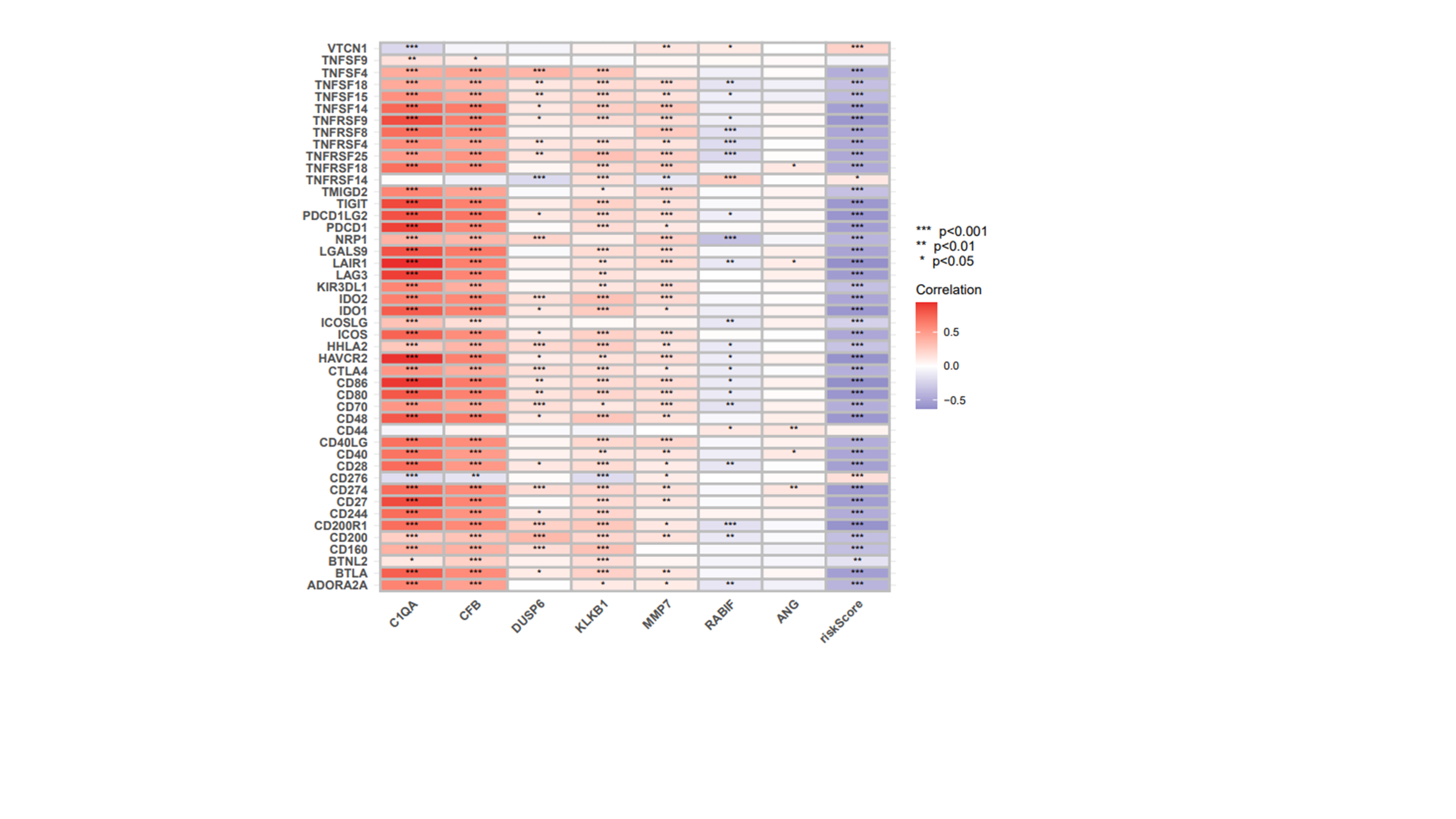
**

**[Supplementary](javascript:;) Figure S1.** Correlation between risk scores and immune checkpoints.


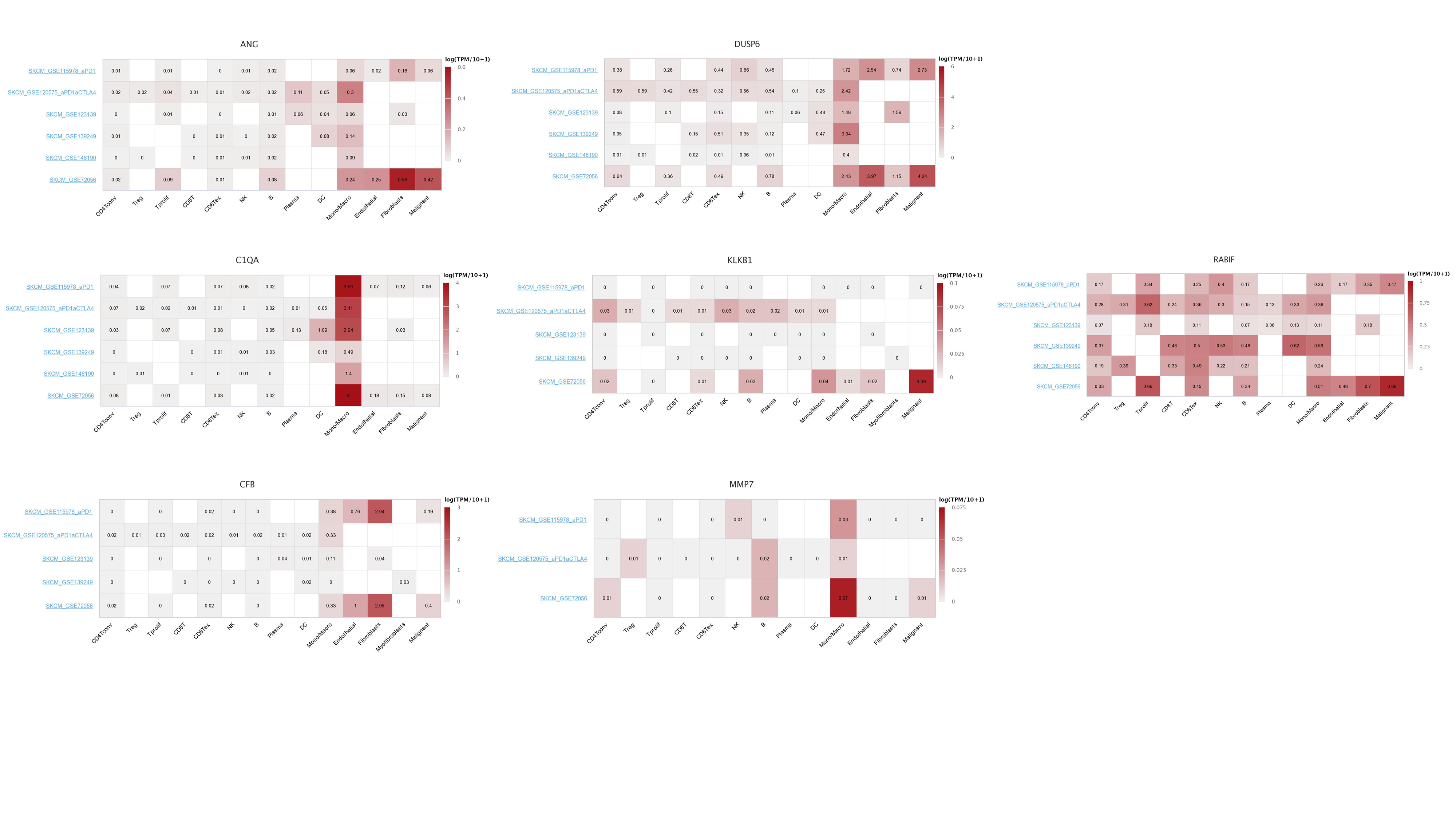


[**Supplementary**](javascript:;) **Figure S2.** Expression levels of CRGs in SKCM TME-associated cells in the GEO database.


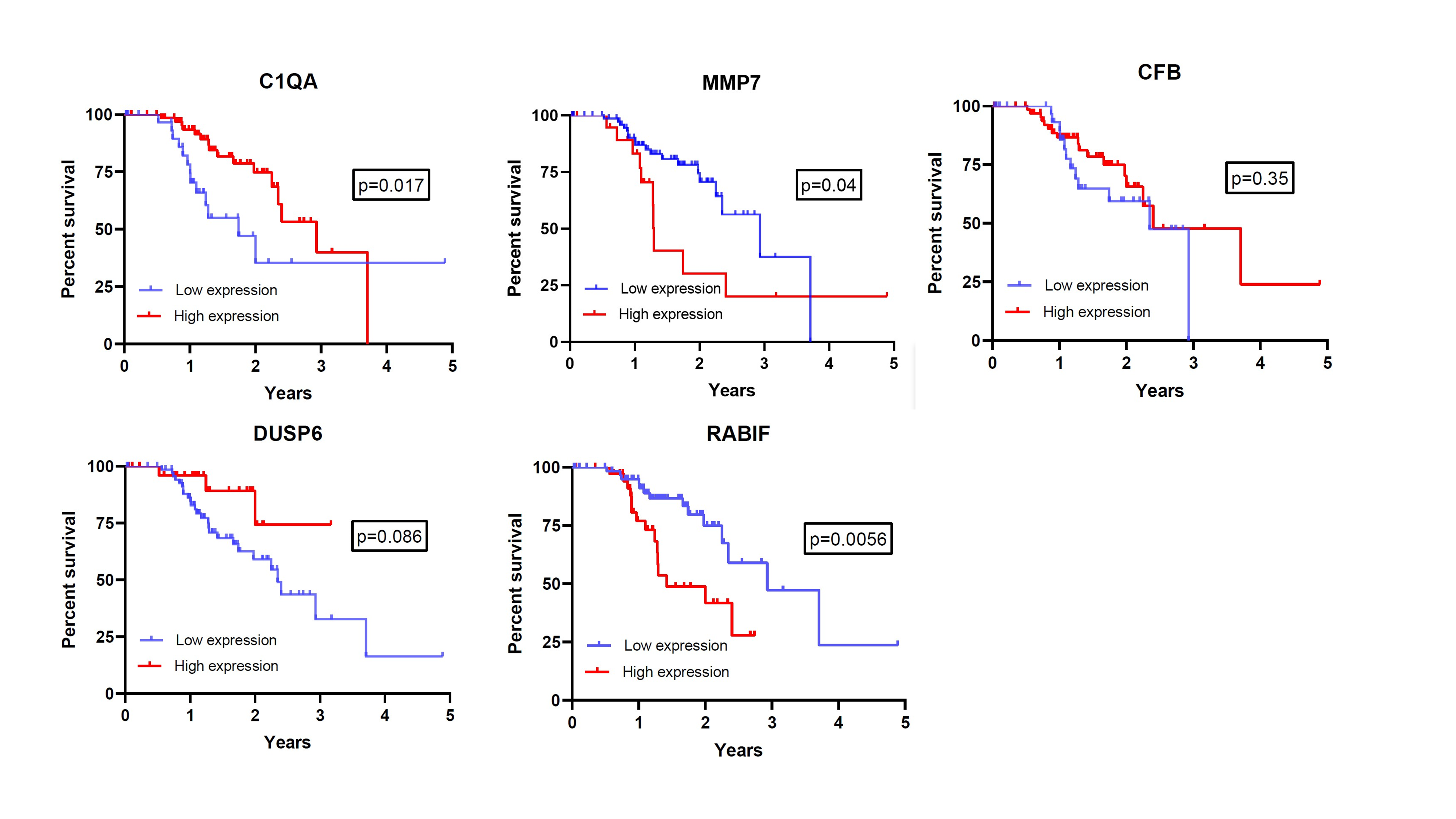


[**Supplementary**](javascript:;) **Figure S3.** KM curves of 5 genes (C1QA, MMP7, CFB, DUSP6, RABIF) in the HPA database
